# Caring Behaviors Inventory-24: translation, cross-cultural adaptation and psychometric testing for use in a Swedish context

**DOI:** 10.1186/s12960-020-00540-5

**Published:** 2021-01-20

**Authors:** Anna Klarare, Mona Söderlund, Anna Wikman, Jenny McGreevy, Elisabet Mattsson, Andreas Rosenblad

**Affiliations:** 1 Department of Health Care Sciences, Ersta Sköndal Bräcke University College, Box 11189, 100 61 Stockholm, Sweden; 2 Department of Women’s and Children’s Health, Clinical Psychology in Healthcare, Uppsala University, Uppsala, Sweden; 3 Department of Women’s and Children’s Health, Reproductive Health, Uppsala University, Uppsala, Sweden; 4 Department of Dietetics, Region Sörmland, Nyköping, Sweden; 5 Centre for Clinical Research Sörmland, Eskilstuna, Sweden; 6 Department of Medical Sciences, Clinical Diabetology and Metabolism, Uppsala University, Uppsala, Sweden

**Keywords:** Caring, Nursing, Translation, Cross-cultural adaptation, Psychometric testing

## Abstract

**Background:**

Patients’ health and wellbeing are promoted when nurses successfully conceptualize caring in clinical practice. Measuring caring behaviors can advance knowledge about caring and has potential to improve caring practices and the outcomes of care. The Caring Behaviors Inventory-24 (CBI-24) is an empirical instrument for measuring caring, developed to determine perceptions of caring among patients and nurses. Since the instrument was not available in Swedish, the aim of this study was therefore to translate into Swedish and cross-culturally adapt CBI-24 for a Swedish healthcare context, and to psychometrically analyze the Swedish version of CBI-24.

**Methods:**

The study used a traditional forward and back translation process in six stages: (1) two simultaneous translations by bilingual experts; (2) expert review committee synthesis; (3) blind back translation; (4) expert review committee deliberations; (5) pre-testing with cognitive interviews, and (6) psychometric evaluations.

**Results:**

The translation process was systematically conducted and entailed discussions regarding semantic, idiomatic, experiential and conceptual equivalence. The cognitive interviews generated thoughts and reflections, which resulted in adjusting three items of the CBI-24 SWE. For psychometric analyses, 234 persons answered the questionnaire. Results indicated acceptable overall model fit in the χ^2^-value for the confirmatory factor analysis, while for the heuristic goodness-of-fit indices, the comparative fit index (CFI) and the standardized mean square residual (SMSR) indicated good model fits, and the root mean square error approximation (RMSEA) indicated an acceptable fit.

**Conclusions:**

CBI-24 SWE has been shown to be a psychometrically acceptable instrument for use in Swedish research contexts. Further studies regarding the clinical usefulness of the instrument may be in order. In particular, CBI-24 SWE should be evaluated among nurses in rural areas.

## Background

Health and wellbeing are promoted when nurses successfully conceptualize caring in clinical practice [1, 2]. The relationship between patient and nurse is regarded as being pivotal to caring practice [3-5] and experiences of quality care [6, 7]. Studying dimensions of caring can be a challenge, especially since caring is a complex phenomenon, and may be regarded as a human trait, a moral imperative, an affect toward something, an interaction and a therapeutic intervention [8]. That caring can be viewed in these diverse ways is one reason for the challenges when studying caring. The dichotomy between *doing* nursing interventions and *being* a caring nurse is debated, especially in light of administration and management of care leaning toward economic considerations [9]. Reducing a complex phenomenon to measurable nursing behaviors entails a risk of losing nuances. However, measuring caring presents opportunities to advance knowledge about caring, and learn more about patients’ and nurses’ experiences to improve caring practices and the outcomes of care [9].

The Caring Behaviors Inventory-24 (CBI-24) is an empirical instrument for measuring caring with a clear conceptual–theoretical basis, developed to determine perceptions of caring among patients and nurses in diverse setting [10, 11]. CBI-24 is extensively used and has shown high internal consistency, convergent validity, and good test–retest reliability when administered to hospitalized patients and nurse [11]. It is, however, not available in Swedish, and to ascertain that an instrument is appropriate and valid for clinical practice and research, validation is imperative [12]. The aim of this study was therefore to translate into Swedish and cross-culturally adapt CBI-24 for a Swedish healthcare context, and to psychometrically analyze the Swedish version of CBI-24 (CBI-24 SWE).

## Methods and material

### The Caring Behaviors Inventory-24 (CBI-24)

The instrument includes four correlated subscales: (I) the Assurance subscale, being readily available to a patient’s needs and security (8 items); (II) the Knowledge and Skill subscale, demonstrating conscience and competence (5 items); (III) the Respectful subscale, attending to the dignity of the person (6 items); and (IV) the Connectedness subscale, providing constant assistance to patients with readiness (5 items). Subjects are asked to rate each item on a six-point Likert scale ranging from 1 (never) to 6 (always). The caring behavior for each subscale as well as for the overall scale is calculated as the mean value within each separate scale [11].

### Design and setting

The project used a traditional forward and back translation process as described by Guillemin, Bombardier and Beaton in 1993 [13], and developed further by Beaton et al [14] in 2000. The process contains six stages: (1) two simultaneous translations by bilingual experts; (2) expert review committee synthesis of the two translations to one; (3) blind back translation to the source language by two new bilingual experts; (4) expert review committee deliberations for consensus on version ready for testing; (5) pre-testing with cognitive interviews, and (6) psychometric evaluations.

The forward translations in the first stage were performed by two native Swedish speakers, one clinically active nurse researcher familiar with the field and one researcher naïve to the field. The second stage synthesis of the two translations was achieved during an expert review committee meeting where the two translations were compared, discussed and reflected on, resulting in one translated version ready for back translation. A person not involved in the translations acted as chairperson and mediator. The third stage back translations were performed by two native English speakers, one naïve to context, originally from North America, and one familiar with clinical work, originally from the United Kingdom. The study was conducted by researchers in university settings, in an urban area in Sweden.

### Expert review committee deliberations

The fourth stage entailed expert review committee deliberations where reports from stages I–III were read, discussed and reflected on. This was a crucial step for cross-cultural adaptation [14] since the processes of forward and back translation may reveal discrepancies and highlight inconsistencies. The expert review committee comprised three translators and two researchers, where one acted as moderator. In this group, there was expertise and competence regarding research, method and language as well as the clinical context through experience as healthcare professionals. Discussion ensued and consensus decisions were made in the four areas of equivalence [14]: semantic (words mean the same, no double meanings), idiomatic equivalence (idioms hard to translate), experiential (daily life experiences) and conceptual (concepts used may differ across cultures and languages). The proceedings and discussions were meticulously documented for transparency and to provide a record of the decisions made.

### Cognitive interviewing

Using cognitive interviewing with the target population has potential to influence reliability and content validity by assessing the clarity and relevance as perceived by the target population [15], in our case women with experiences of homelessness, nursing students and registered nurses. Cognitive interviews, using a “think aloud” approach [12] to explore face and content validity, were performed with women having experiences of homelessness (*n* = 5), nursing students (*n* = 5) and registered nurses (*n* = 5). Participants were purposively recruited to meet the inclusion criteria of either being a woman with experience of homelessness, a nursing student or a registered nurse interacting with persons in homelessness in their clinical work. Women were chosen since the CBI-24 will be used in a larger project focusing on women in homelessness. Participants were encouraged to fill out the questionnaire while thinking out loud and to give words to the thoughts going through their minds during the process. Probes were used by the interviewer to elicit further information regarding thoughts about questionnaire items, instructions, and response options. The interviews were audio recorded with the participant’s permission. A small voucher, valid in grocery stores or movie theaters, was offered at interview conclusion in appreciation of participation.

For analysis, interviews were listened to and participants’ thoughts were documented in a template in writing, first one interview at a time, and secondly compiled as a comprehensive summary of the whole. The expert review committee convened to discuss outcomes and suggested minor revisions of wording or sentence structure in a few questionnaire items.

### Data and setting for the psychometric analysis

For the psychometric analysis of CBI-24 SWE, registered nurses and nurse students were asked to participate in the study by answering an anonymous online questionnaire containing the CBI-24 questions as well as some questions about background characteristics. Participants were approached face-to-face by researchers, in two clinical units and one university setting. In total, 234 individuals answered the questionnaire during October and November 2019.

### Statistical analyses

Categorical data are given as frequencies and percentages, *n* (%), while ordinal and continuous data are presented as means and standard deviations (SDs). In the psychometric analysis, the main interest was to examine if the hypothesized CBI-24 factor structure fitted the collected data for the CBI-24 SWE. To this end, a confirmatory factor analysis (CFA) was applied, using a χ^2^-test to assess the overall model fit. A normed χ^2^-value < 5.0 was considered to indicate an acceptable model fit. The goodness-of-fit indices Comparative Fit Index (CFI), Standardized Root Mean Square Residual (SRMR), and Root Mean Square Error of Approximation (RMSEA) were used as heuristic measures of model fit. Values of CFI > 0.90 and SRMR < 0.08 were considered to indicate good model fits, while values of RMSEA ≤ 0.05 and RMSEA < 0.08 were considered as close and acceptable model fits, respectively. [16, 17] An item reliability *R*^2^ ≥ 0.40 was deemed acceptable. The R package ‘lavaan’ version 0.6–5 (Rosseel, 2012) was used for the CFA analyses, applying the full information maximum likelihood (FIML) estimator to handle potentially missing data. All statistical analyses were performed in R 3.6.1 (R Foundation for Statistical Computing, Vienna, Austria), with *P*-values < 0.05 considered statistically significant.

All proceedings adhered to ethical principles outlined by the World Medical Association, [18] and the study had ethical approval from the Swedish Ethical Review Authority.

## Results

### Translations, cultural adaptation and expert review committee discussions

The translation process was systematically conducted and entailed discussions regarding semantic, idiomatic, experiential and conceptual equivalence throughout. Experiences from forward and back translations indicated that most questions were straightforward and thus easily translated. For these questions, back translation did not reveal any discrepancies and translations of the questions were easily agreed on. A few questions were more challenging. Question number 7 (*helping the patient grow*) was tricky since the idiomatic character of the statement may be difficult to word equivalently in Swedish. After expert review committee discussions, a suggestion including a cultural adaptation resulted in a Swedish wording in terms of ‘helping the patient develop as a person’ (hjälper patienten att utvecklas som person). Question number 8 (*being patient or tireless with the patient*) was also discussed since finding equivalent wording in Swedish was challenging, and the question implicitly presented two concepts (patience and endurance). The sentence was somewhat differently constructed in Swedish, aiming for conceptual equivalence, stating ‘shows patience with the patient, without getting tired’ (visar tålamod med patienten, utan att tröttna). The third question that was discussed in detail was question number 22 (*showing concern for the patient*). There were discussions pertaining to semantic equivalence; does concern have positive or negative connotations, i.e., is the nurse concerned because something is wrong, as with a risk of some kind, or is the nurse concerned for the patient as an expression of caring? Since CBI-24 has a caring focus, the expert group decided to interpret the statement as positive and consequently the Swedish statement reads ‘cares for the patient’ (bryr sig om patienten). The stages of translation and expert review committee discussions resulted in a Swedish version ready for initial testing through cognitive interviewing with the target populations, namely women with experiences of homelessness, registered nurses and nurse students.

### Cognitive interviewing

The cognitive interviews generated thoughts and reflections on the CBI-24 SWE questionnaire, with consensus being that the questions were relevant and understandable, thus establishing face validity. Participants (n = 15) stated that some questions were easy to answer, while others required some reflection. Three questions were adjusted because of feedback from the cognitive interviews: Question 15 (*treating patient information confidentially*) was unclear to most participants; more specifically, the word confidential was hard for registered nurses, nurse students and women with experiences of homelessness to understand. In the Swedish version, the question was changed to ‘treating patient information so that unauthorized persons cannot access it’ (hanterar information om patienten så att obehöriga inte kan ta del av den). Regarding question 16 (*returning to the patient voluntarily*), there were several participants who wondered why the nurse should return? In the Swedish question, the wording was changed by adding ‘if there is a need’ (om behov finns, återkommer självmant till patienten). The third question that was changed after the cognitive interviews was question 20 (*responding quickly to the patient’s call*). Participants found the question confusing and unclear, since they felt it was not stated whether the call was by telephone, ward call button or in some other way. Therefore, the question was revised to read: ‘responds quickly when the patients call for help, for example by ward call button or telephone’ (agerar snabbt när patienten vill ha hjälp, tex. ringer på larmklocka eller telefon). Having preliminarily established face and content validity through the cognitive interviews, CBI-24 SWE was considered ready for a formal psychometric analysis.

### Psychometric analysis

Background characteristics for the 234 individuals who answered the CBI-24 SWE questions are given in Table 1. Of the participants, 92 (39.3%) were nurses and 142 (60.7%) nurse students. On average, the nurses were 14 years older than the nurse students, 45.2 compared to 31.2 years old. Most participants, 83 (91.2%) nurses and 131 (92.3%) nurse students, identified as female. Among the nurses, 59 (64.1%) had worked more than 10 years in the profession, while among nurse students, being in the 3^rd^ (n = 42; 29.6%) or 6^th^ (n = 39; 27.5%) semester were the most common answers.

**Table 1:** Background characteristics of participants included in psychometric analysis (n = 234)

Variable	Nurses n = 92		Nurse students n = 142
Age, mean (SD)	45.2 (11.3)		31.2 (7.9)
Female gender identification, n (%)	83 (91.2)		131 (92.3)
Years in profession, n (%)		Semester, n (%)	
< 1 year	1 (1.1)	3^rd^	42 (29.6)
1–2 years	1 (1.1)	4^th^	33 (23.2)
3–5 years	10 (10.9)	5^th^	28 (19.7)
6–10 years	21 (22.8)	6^th^	39 (27.5)
> 10 years	59 (64.1)		

In the CFA analysis, the χ^2^-value for the CBI-24 SWE was 538.669 with 246 degrees of freedom (P < 0.001), resulting in a normed χ^2^-value of 2.19, indicating an acceptable overall model fit. For the heuristic goodness-of-fit indices, the resulting values of CFI = 0.910 and SRMR = 0.065 also indicated good model fits. However, the RMSEA value of 0.071 (95% CI 0.063–0.079), with P < 0.001 for testing of the null hypothesis RMSEA ≤ 0.05 (i.e., a close fit), did not indicate a close model fit, but only an acceptable fit.

Table 2 gives factor loadings and item reliability for CBI-24 SWE. For the present data set, Assurance had the strongest influence on the variable Q17 (“talking with the patient”), with each unit increase in Assurance implying a 0.82-unit increase in Q17 (P < 0.001), while Knowledge and skill had the strongest influence on the variable Q11 (“demonstrating professional knowledge and skill”), with a 0.69-unit increase in Q11 for each unit increase in Knowledge and skill (P < 0.001). The dimension Respectful had the strongest influence on the variable Q13 (“allowing the patient to express feelings about his or her disease and treatment”), with each unit increase in Respectful giving a 0.85-unit increase in Q13 (P < 0.001), while the dimension Connectedness had the strongest influence on the variable Q7 (“helping the patient grow”), with each unit increase in Connectedness implying a 0.97-unit increase in Q7 (P < 0.001).

**Table 2:** Factor loadings and item reliability for the Caring Behaviors Inventory-24 (CBI-24 SWE) instrument

Factor	Indicator	Factor loadings			Standardized estimate	Item reliability (*R*^2^)
		Estimate	95% CI	*P*-value		
Assurance	Q16	0.74	0.62–0.86	< 0.001	0.71	0.505
	Q17	0.82	0.70–0.93	< 0.001	0.77	0.588
	Q18	0.60	0.49–0.70	< 0.001	0.67	0.442
	Q20	0.63	0.51–0.74	< 0.001	0.64	0.414
	Q21	0.40	0.31–0.50	< 0.001	0.51	0.263
	Q22	0.64	0.55–0.73	< 0.001	0.77	0.600
	Q23	0.32	0.22–0.41	< 0.001	0.44	0.192
	Q24	0.41	0.33–0.50	< 0.001	0.57	0.327
Knowledge and skill	Q9	0.52	0.43–0.61	< 0.001	0.67	0.443
	Q10	0.56	0.46–0.66	< 0.001	0.68	0.459
	Q11	0.69	0.61–0.77	< 0.001	0.89	0.785
	Q12	0.62	0.52–0.72	< 0.001	0.72	0.519
	Q15	0.41	0.28–0.54	< 0.001	0.42	0.175
Respectful	Q1	0.68	0.59–0.77	< 0.001	0.80	0.639
	Q3	0.70	0.60–0.80	< 0.001	0.78	0.616
	Q5	0.78	0.67–0.89	< 0.001	0.78	0.614
	Q6	0.77	0.67–0.88	< 0.001	0.79	0.622
	Q13	0.85	0.74–0.96	< 0.001	0.80	0.646
	Q19	0.60	0.50–0.70	< 0.001	0.68	0.468
Connectedness	Q2	0.62	0.50–0.74	< 0.001	0.61	0.367
	Q4	0.93	0.80–1.06	< 0.001	0.77	0.593
	Q7	0.97	0.83–1.11	< 0.001	0.76	0.583
	Q8	0.82	0.71–0.93	< 0.001	0.81	0.654
	Q14	0.82	0.70–0.94	< 0.001	0.75	0.562

The item reliabilities (*R*^2^-values) for the 24 items in the CBI-24 instrument are also given in Table 2. While most of these values were ≥ 0.40 and thus deemed acceptable, the item reliabilities for questions Q2 (“giving instructions or teaching the patient”), Q15 (“treating patient information confidentially”), Q21 (“helping to reduce the patient’s pain”), Q23 (“giving the patient’s treatments and medications on time”), and Q24 (“relieving the patient’s symptoms”) were all ≤ 0.367, implying a questionable item reliability. In particular, questions Q15 and Q23 had low item reliabilities, with *R*^2^-values of 0.175 and 0.192, respectively, suggesting that CBI-24 SWE may benefit from a reduction of the number of items by removing questions Q15 and Q23.

## Discussion

In this study, CBI-24 was translated into Swedish, culturally adapted and psychometrically analyzed. Semantic, idiomatic, experiential and conceptual equivalence [14] was strived for in translation proceedings and, furthermore, explored in cognitive interviews with women with experiences of homelessness, registered nurses and student nurses. We demonstrated acceptable overall model fit in the χ^2^-value for the CFA, while for the heuristic goodness-of-fit indices, the CFI and the SRMR indicated good model fits, and the RMSEA indicated an acceptable fit. These measures have led to an acceptable and ready-to-use CBI-24 SWE for research purposes in Swedish healthcare contexts.

One of the benefits of translating and adapting existing questionnaires is that it allows comparisons across countries and populations [12]. Our findings support that, when attempting to prepare a research instrument for use in another language, merely translating the words is insufficient, since nuances demand various forms of interpretation that entail subsequent decisions and actions [19, 20]. A systematic approach is crucial to promote content validity and reliability [15]. In the process described by Beaton et al. [14], experts are involved in different stages, contributing to the development of the instrument in the target language. Using different types of experts has the potential to provide a fuller understanding of how the research instrument is accepted and how different forms of input can inform development further [15]. The experts provided input with bearing on the four aspects of equivalence: semantic, idiomatic, experiential and conceptual [14]. Semantic and idiomatic aspects were discussed at all stages of the translation and cultural adaptation. Experiential equivalence was highlighted in the interviews and the analyses, while conceptual equivalence was discussed in depth in multiple expert review committee discussions. Describing the process and the analyses clearly has bearing on the trustworthiness of the final version of the instrument [21].

Caring has been advocated as the core of nursing and is a multifaceted phenomenon [1, 4, 8]. Building a relationship has been highlighted as crucial for caring practice [3, 5], and the character of the nurse–patient relationship is associated with experiences of quality care [7]. However, patients’ and nurses’ views of caring behaviors may be aligned or disparate and this warrants further exploration [22, 23]. *Doing* nursing interventions or *being* a nurse has been investigated by Watson [9], and it is likely that nurses’ professional approach will be affected depending on how the individual nurse interprets and operationalizes *doing* nursing or *being* a nurse. Similarly, patients and families have expectations of nursing interventions and caring, meeting these expectations, or not, will probably also affect experiences of care quality [24-26]. Using CBI-24 and analyzing disparate views of stakeholders can be one way forward to promote the development of healthcare toward less authoritarian structures, focusing on meeting patients’ needs and promoting caring behaviors beneficial to health and wellbeing.

### Strengths and limitations

A limitation of the present study was that only nurses in urban settings were recruited to the study, since it is possible that nurses working in rural areas may have answered some of the questions differently. Another limitation is that only women in homelessness were included for cognitive interviewing, it is possible that opinions from other genders would differ. Moreover, although the sample size of *n* = 234 available observations for the psychometric analysis was above the commonly recommended minimum sample size of *n* = 200 [16, 17], considering the number of items in the CBI-24 instrument, it may have been beneficial to aim for a sample size of at least *n* = 300, in order to obtain more stable results.

## Conclusions

CBI-24 SWE has been shown to be a psychometrically acceptable instrument for use in Swedish research contexts. The translation and cultural adaptation processes involved experts who made significant contributions by providing perspective at the different stages. The systematic approach, comprising both quantitative and qualitative design, enabled diverse expert input to inform the shaping and construction of CBI-24 SWE, which improved content validity and reliability. Further studies regarding the clinical usefulness of the instrument may be in order. In particular, CBI-24 SWE should be evaluated among nurses in rural areas.

## Data Availability

The datasets used and/or analyzed during the current study are available from the corresponding author on reasonable request.

## Abbreviations

CBI-24: Caring Behaviors Inventory- 24
CBI-24 SWE: Caring Behaviors Inventory- 24, Swedish version
CFA: Confirmatory factor analysis
CFI: Comparative Fit Index
FIML: Full information maximum likelihood
RMSEA: Root mean square error of approximation
SRMR: Standardized mean square residual
